# Factors Influencing Unmet Healthcare Needs among Older Korean Women

**DOI:** 10.3390/ijerph18136862

**Published:** 2021-06-26

**Authors:** Jung A. Choi, Oksoo Kim

**Affiliations:** 1 Ewha Research Institute of Nursing Science, College of Nursing, Ewha Womans University, Seoul 03760, Korea; jungah.ch@gmail.com; 2 College of Nursing, Ewha Womans University, Seoul 03760, Korea

**Keywords:** unmet healthcare needs, older women, Korean, residential area

## Abstract

The purpose of this study was to determine factors that influence the unmet healthcare needs of older women in Korea and to examine differences in the reasons for these unmet healthcare needs according to age and residential area. We analyzed data from the 2018 Korea Community Health Survey and enrolled 42,698 older Korean women in this study. Residential area, living arrangement, income, education, basic livelihood subsidy, activity of daily living, subjective health status, hypertension and diabetes, unmet healthcare needs, and the reasons healthcare needs were not met were assessed. Logistic regression analysis was performed to identify factors that influenced unmet healthcare needs. Chi-square tests were used to identify reasons for unmet healthcare needs according to age group and residential area. Of the participants, 4151 (9.7%) reported unmet healthcare needs over the past year. The primary reason participants could not use health services was “inconvenient transportation” (38.4%), followed by “financial burden” (28.4%) and “symptoms not severe” (16.8%). There were significant differences in “financial burden”, “difficulty making appointments”, “inconvenient transportation”, and “symptoms not severe” according to both age group and residential area. Factors that influenced unmet healthcare needs were residential area, living alone, lower family income, lower educational level, basic livelihood subsidy, difficult activities of daily living, hypertension and diabetes, and poor subjective health. Older women in Korea living alone in urban and rural areas had more unmet healthcare needs of than those who lived with other people. To address the unmet healthcare needs of older Korean women, transportation and medical facilities need to be improved or established.

## 1. Introduction

The population aged 65 or older accounted for 14.9% of the total population of Korea in 2019, and this population is expected to increase to 33.9% in 2040 [1]. The prevalence of chronic diseases is high among older adults, with 64.1% of men and 79.5% of women reporting multiple chronic conditions [2]. Older adults are a vulnerable group with a higher percentage of unmet health care needs than younger or middle-aged adults [3].

Compared to older men, older Korean women not only have lower levels of education and economic status [4], but also a lower subjective health level and health-related quality of life [5,6,7]. In addition, older women in Korea find it more difficult to maintain activities of daily living than older men [8,9]. These health and socio-economic deficits of older women may prevent them from maintaining optimal health levels and accessing necessary medical services. Ultimately, these deficits may shorten the healthy lifespan of older Korean women and increase disease prevalence and mortality [10]. These health disparities therefore need to be resolved.

Unmet healthcare needs (UHNs) refer to the gap between the health care services required to deal with health problems and the health care services actually used, and are a measure of medical services accessibility [11]. Access to health care services is an important factor that determines level of health, and ensures equitable access to health care resources and is one of the most important goals of health care systems [12]. In 2018, the average rate of UHNs in EU countries was 3.2%, and in 2016, 7–33% of the populations of 11 countries were reported to have UHNs [13]. In 2016, 8.8% of the population of Korea reported UHNs, which indicates that barriers to the use of medical services is not high. However, the income of older adults in Korea is in the bottom 20% and the rate of their use of medical services is more than twice that of older adults in the top 20% of income [14]. This means that the gap between groups of older adults in terms of unmet health care needs is large.

Previous studies have reported that age, gender, low income, and low education level are related to unmet healthcare needs [15,16,17,18]. Previous studies have confirmed relationships between socioeconomic status, health status, and UHNs [19,20,21]. However, there is a lack of studies that have examined regional differences in UHNs and causes for these UHNs such as availability and accessibility of medical services. The characteristics of the residential area (urban vs. rural vs. metropolitan) can affect the use of medical services, resulting in differences in the health of older adults [22,23,24,25]. Therefore, it is necessary to consider differences in residential area to analyze older adults’ use of medical services and factors that influence this.

Our aims in this study were to analyze factors influencing unmet healthcare needs in older women in Korea and the reasons why there were unmet healthcare needs according to age and residential area using data from the 2018 Korea National Community Health Survey. Our findings can be used in policy development to improve equity in the use of health services by older women.

## 2. Materials and Methods

### 2.1. Data Source and Samples

In this study, data from the 2018 Korea Community Health Survey (KCHS) conducted by the Korea Centers for Disease Control and Prevention were analyzed. The KCHS used a stratified two-stage cluster sampling method. Sample residential areas were selected using a stratified cluster sampling method and sample households were selected using a systematic sampling method. All household members aged 19 years and older in the sample households were selected as the target population. Trained investigators visited selected sample households and collected data through interviews from August to October in 2018. In this study, out of 228,340 participants in the 2018 KCHS, 72,492 older adults over 65 years of age were extracted, of which 42,698 older women were targeted.

### 2.2. Instruments

#### 2.2.1. Baseline Characteristics

Baseline characteristics included age, residential area, living arrangement, family income, education, basic livelihood subsidy, activities of daily living, diagnoses of hypertension and diabetes, and subjective health. Residential areas were divided into metropolitan, urban, and rural areas. Participants were asked whether they were receiving a basic livelihood subsidy from the government using a ‘yes’ or ‘no’ question format. Activities of daily living were assessed as being ‘easy’, ‘a little difficult’, or ‘difficult’ to fulfill. Participants were asked if they had ever been diagnosed with hypertension and diabetes. Subjective health was measured as “good”, “fair”, or “poor”.

#### 2.2.2. Unmet Healthcare Needs

To investigate whether subjects experienced UHNs, participants were asked to respond with “yes” or “no” to the following question: “During the past year, have you ever been unable to go to a hospital or clinic (not including dentistry) when you wanted to go to there?” The reason the participant did not go to the hospital or clinic was determined based on their response to the following options: “symptoms not severe”, “long waits for appointments”, “inconvenient time”, “inconvenient transportation”, “difficulty making appointments”, or “financial burden”.

### 2.3. Statistical Analysis

Data analysis was performed by reflecting weights using SPSS version 25 (IBM, Armonk, NY, USA). The significance of associations between background variables and UHNs were analyzed using χ^2^-tests and *t*-tests. The significance of differences in UHNs according to age group and residential area were analyzed using χ^2^-tests. Logistic regression analysis was performed to identify factors influencing UHNs. Statistical significance was declared if *p* value <0.05.

### 2.4. Ethical Considerations

This study was conducted after approval was received from the Korea Centers for Disease Control and Prevention for the use of KCHS data. In addition, the study received confirmation of exemption from deliberation by the Institutional Review Board of the University (IRB No. ewha-202004-0033-01) for secondary analysis of KCHS data. The KCHS data are available in the public domain and do not include personal information.

## 3. Results

Of the participants, 4151 (9.7%) reported a UHN over the past year. Whether or not there was a UHN differed significantly according to age, residential area, living arrangement, family income, education, basic livelihood subsidy, activities of daily living, and subjective health. There were no significant differences in UHNs according to the diagnoses of hypertension and diabetes (Table 1).

**Figure 1:**
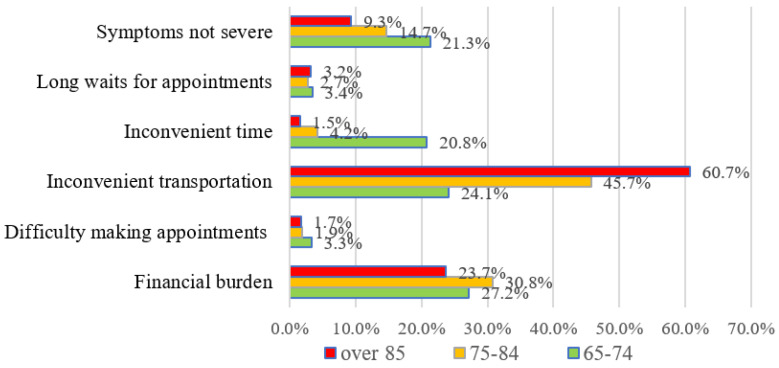
Reasons healthcare needs were not met according to age group.

**Figure 2:**
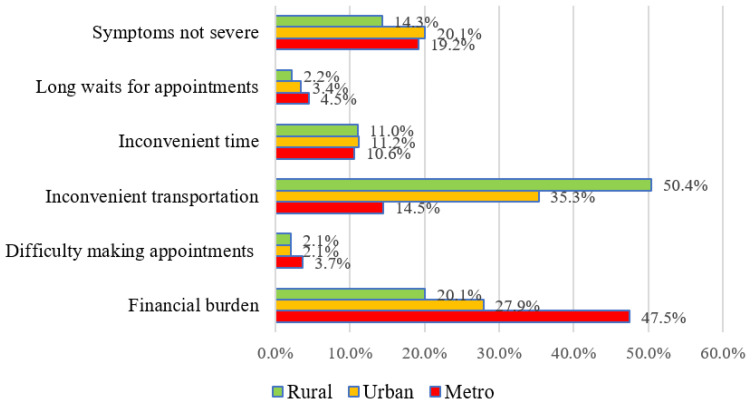
Reasons healthcare needs were not met according to residential area.

A total of 3334 of the 4151 participants provided a reason for UHNs, and the results are presented in Table 2 and Figure 1 and Figure 2. The highest frequency reason why participants did not use health services was “inconvenient transportation” (38.4%) followed by “financial burden” (28.4%) and “symptoms not severe” (16.8%).

The results of analysis of the reasons for unmet healthcare needs by age group and residential area are presented in Figure 1 and Figure 2. The most frequent reason why healthcare needs were unmet in participants aged 65 to 74 was “financial burden” (27.2%), followed by “inconvenient transportation” (24.1%) and “symptoms not severe” (21.3%). The most frequent reason for participants aged 75 to 84 and over 85 was “inconvenient transportation” (45.7% and 60.7%, respectively) followed by “financial burden” (30.8% and 23.7%, respectively) and “symptoms not severe” (14.7% and 9.3%, respectively). When UHNs were analyzed according to residential area, the most frequent reason for UHNs of metropolitan residents was “financial burden” (47.5%), followed by “symptoms not severe” (19.2%) and “inconvenient transportation” (14.5%). For both urban and rural residents, “inconvenient transportation” was the most common reason for UHNs at 35.3% and 50.4%, respectively, followed by “financial burden” at 27.9% and 20.1%, respectively, and “symptoms not severe” at 20.1% and 14.3%, respectively.

There were significant differences in “financial burden”, “difficulty making appointments”, “inconvenient transportation”, and “symptoms not severe” according to both age group and residential area. There was a significant difference in “inconvenient time” according to age group (Table 2).

Table 3 shows the factors influencing UHNs. Hosmer and Lemeshow tests were used for model fitting (χ^2^ = 8.97, *p* = *0*.345). The variance inflation factors (VIFs) were between 1.03 and 1.17 and indicated absence of multicollinearity problem. The classification accuracy of the logistic regression model was 64.3% and the explanatory power of the regression model was 7.8%. Factors influencing UHNs were residential area, living arrangement, family income, education, basic livelihood subsidy, activities of daily living, diagnoses of hypertension and diabetes, and subjective health. Likelihood of experiencing UHNs was higher for residents of urban (OR = 1.18, 95% CI, 1.08–1.28) and rural areas (OR = 1.34, 95% CI, 1.21–1.47) than residents of metropolitan areas. Participants living alone experienced more UHNs than those living with others (OR = 1.21, 95% CI, 1.13–1.31), and those with an education level of elementary school or lower (OR = 1.73, 95% CI, 1.27–2.36) or high school (OR = 1.40, 95% CI = 1.02–1.92) also experienced more UHNs than those with college or higher education. The likelihood of UHNs was lower for participants with a monthly family income of 2000–4000 US dollars (OR = 0.71, 95% CI, 0.62–0.81) and 4000–6000 dollars (OR = 0.54, 95% CI, 0.42–0.70) than those of participants with a monthly income of 6000 dollars or more. Participants who received a basic livelihood subsidy (OR = 1.67, 95% CI, 1.50–1.85) had a higher likelihood of UHNs than those who did not. Participants who reported difficulty with ADLs (OR = 3.43, 95% CI, 2.91–3.96) or a little difficulty with ADLs (OR = 1.81, 95% CI, 1.68–1.95) were more likely to have UHNs than those who reported no difficulties with ADLs. Participants whose subjective health status was fair (OR = 0.60, 95% CI, 0.55–0.65) or good (OR = 0.45, 95% CI, 0.40–0.51) were less likely to have UHNs than those who reported a poor health status. Participants diagnosed with hypertension (OR = 0.87, 95% CI, 0.80–0.94) or diabetes (OR = 0.88, 95% CI, 0.81–0.95) were less likely to have UHNs than those who did not have these diagnoses.

## 4. Discussion

In this study, we analyzed the UHNs of older women in Korea and reasons for these UHNs. In this study, 9.7% of the participants had UHNs. This is higher than the average reported for EU countries of 3.2%, 13 but lower than the 14.0% reported for OECD countries. The proportion of UHNs varies from country to country, with values of 19% reported for Estonia, 7.0% for the UK and Sweden, 6.0% for Germany, and 8.0% for Norway [26].

In our study, living alone, receiving a basic living subsidy, a lower level of education, and poor health were factors that increased the likelihood of UHNs, consistent with previous studies [20,27]. Older adults living alone have a higher prevalence of chronic diseases than those living with their spouses or children, in addition to more functional limitations [2]. Older women in Korea are more vulnerable to health care deficits because of their higher prevalence of chronic diseases and lower educational and economical levels than older men [28,29]. In addition, because living alone increases the likelihood that older women will experience UHNs, older women living alone are at elevated risk of having unmet healthcare needs. As the life expectancy of older adult increases, the state of living alone can be prolonged, and the prevalence and complications of chronic diseases will likely increase, making it necessary to develop policies and provide a support system for older women living alone. In this study, a low level of education and a basic living subsidy were positively associated with UHNs, similar to what has been reported for poor people in other countries [13].

In this study, family income and basic living subsidy were used as indicators of the financial status of participants. We found that a high family income and a basic living subsidy increased the likelihood of UHNs. However, it is the basic living subsidy rather than family income that directly reflects a participant’s economic status. We interpret our findings to mean that low economic status increases the likelihood of UHNs. Efforts are needed to improve the access of low-income older women living alone to medical services.

We found that financial burden, inconvenient transportation, and the notion that the symptoms were not serious were factors positively associated with UHNs. A previous study reported that financial burden is a major impediment to the use of medical services [3]. It was reported that non-regular wage workers had a higher ratio of UHNs than regular wage workers due to the economic burden experienced by the former [24]. In OECD countries, the ratio of UHNs among upper-income older adults was 8–9%, whereas that among lower-income older adults was as high as 27% [26]. This shows that support for older adults with low income levels should be considered to address UHNs.

Inconvenient transportation is also a major impediment to medical facility access, and transportation barriers have a greater impact on rural residents and low-income families than urban or metropolitan residents and high-income families [30]. In this study, because the proportion of participants residing in rural areas was high, we were not surprised by our finding that transportation inconvenience was a major factor impeding access to healthcare and therefore UHNs.

Factors influencing unmet healthcare needs differed according to where the participant lived and their age group. For older women living in large cities, financial burden was the most important factor, while for older women living in urban and rural areas, inconvenient transportation was the main factor. These findings suggest that urban and rural areas lack public transportation options compared to metropolitan cities. Considering that the proportion of older women in rural areas is higher than in metropolitan cities, and that transportation in rural areas is limited, support for transportation is very important to ensure that older adults in rural areas with chronic diseases are able to access healthcare facilities. In this study, we confirmed that inconvenient transportation was a major barrier to the use of medical services by older women. Inconvenient transportation was the main factor affecting UHNs in older adults over 75 years of age, indicating that as age increases, the means used to access medical services is an important determinant of UHNs.

## 5. Conclusions

In this study, we analyzed factors influencing the UHNs of older women in Korea. By analyzing differences in factors hindering the use of medical services according to age and residential area, we were able to make policy development recommendations to improve health equity. Older women living alone and those living urban and rural areas had more UHNs than older women living with other people and those living in metropolitan areas. To address the UHNs of this population, it is necessary to improve infrastructure systems such as transportation and medical facilities.

## Figures and Tables

**Table 1:** Baseline characteristics by unmet healthcare needs.

Variables	*n* (%)	Unmet Healthcare Needs		χ^2^	*p*
		Yes (*n* = 4151)	No (*n* = 38,547)		
		*n* (%)	*n* (%)		
Age					
65~74	21,135(49.5)	1715(41.3)	19,420(50.4)	154.64	<0.001
75~84	17,618(41.5)	1896(45.7)	15,722(40.8)		
≥85	3945(9.2)	540(13.0)	3405(8.8)		
Residential area					
Metropolitan	13,846(32.4)	1025(24.7)	12,821(33.3)	127.12	<0.001
Urban	8786(20.6)	981(23.6)	7805(20.2)		
Rural	20,066(47.0)	2145(51.7)	17,921(46.5)		
Living arrangement					
Living alone	15,748(36.9)	1881(45.3)	13,867(36.0)	140.43	<0.001
Living with others	26,950(63.1)	2270(54.7)	24,680(64.0)		
Family income (monthly) (US dollars)					
<2000	30,062(70.4)	3148(75.8)	26,914(69.8)	128.83	<0.001
2000–4000	4512(10.6)	284(6.8)	4228(11.0)		
4001–6000	1646(3.9)	76(1.8)	1570(4.1)		
>6000	6478(15.2)	643(15.5)	5835(15.1)		
Education (*n* = 42,641)					
≤Elementary school	32,810(76.9)	3554(85.7)	29,256(76.0)	208.69	<0.001
High school	8590(20.2)	547(13.2)	8043(20.9)		
≥College	1241(2.9)	45(1.1)	1196(3.1)		
Basic livelihood subsidy (*n* = 42,656)					
Yes	2947(6.9)	523(12.6)	2424(6.3)	234.15	<0.001
No	39,709(93.1)	3614(87.4)	36,095(93.7)		
Activities of daily living (*n* = 42,696)					
Easy	26,377(61.8)	1679(40.5)	24,698(64.1)	1075.2	<0.001
A little difficult	14,975(35.1)	2124(51.2)	12,851(33.3)		
Difficult	1344(3.1)	347(8.4)	997(2.6)		
Diagnosed with hypertension (*n* = 42,675)					
Yes	25,707(60.3)	2534(61.1)	23,173(60.1)	1.34	0.249
No/	16,968(39.7)	1615(38.9)	15,353(39.9)		
Diagnosed with diabetes (*n* = 42,670)					
Yes	8878(20.8)	858(20.7)	8020(20.8)	0.04	0.855
No	33,792(79.2)	3289(79.3)	30,503(79.2)		
Subjective health (*n* = 42,685)					
Good	7287(17.1)	354(8.5)	6933(18.0)	749.86	<0.001
Fair	15,971(37.4)	1084(26.1)	14,887(38.6)		
Poor	19,427(45.5)	2711(65.3)	16,716(43.4)		

**Table 2:** Differences in reasons for unmet healthcare needs according to age and residential area.

Categories	*n* (%)		Age			χ^2^ (*p*)	Residential Area			χ^2^ (*p*)
			65~74	75~84	≥85		Metropolitan	Urban	Rural	
			(*n* = 1416)	(*n* = 1508)	(*n* = 410)		(*n* = 785)	(*n* = 796)	(*n* = 1753)	
			*n* (%)				*n* (%)			
Financial burden	947 (28.4)	Y	385	465	97	9.95 (0.007)	373	222	352	200.83 (<0.001)
			(40.7)	(49.1)	(10.2)		(39.4)	(23.4)	(37.2)	
		*N*	1031	1043	313		412	574	1401	
			(43.2)	(43.7)	(13.1)		(17.3)	(24.0)	(58.7)	
Difficulty making appointments	82 (2.5)	Y	47	28	7	7.61 (0.022)	29	17	36	6.54 (0.038)
			(57.3)	(34.1)	(8.5)		(35.4)	(20.7)	(43.9)	
		*N*	1369	1480	403		756	779	1717	
			(42.1)	(45.5)	(12.4)		(23.2)	(24.0)	(52.8)	
Inconvenient transportation	1279 (38.4)	Y	341	689	249	243.12 (<0.001)	114	281	884	299.76 (<0.001)
			(26.7)	(53.9)	(19.5)		(8.9)	(22.0)	(69.1)	
		*N*	1075	819	161		671	515	869	
			(52.3)	(39.9)	(7.8)		(32.7)	(25.1)	(42.3)	
Inconvenient time	364 (10.9)	Y	294	64	6	247.85 (<0.001)	83	89	192	0.15 (0.926)
			(80.8)	(17.6)	(1.6)		(22.8)	(24.5)	(52.7)	
		*N*	1122	1444	404		702	707	1561	
			(37.8)	(48.6)	(13.6)		(23.6)	(23.8)	(52.6)	
Long waits for appointments	101 (3.0)	Y	48	40	13	1.38 (0.501)	35	27	39	9.67 (0.008)
			(47.5)	(39.6)	(12.9)		(34.7)	(26.7)	(38.6)	
		*N*	1368	1468	397		750	769	1714	
			(42.3)	(45.4)	(12.3)		(23.2)	(23.8)	(53.0)	
Symptoms not severe	561 (16.8)	Y	301	222	38	41.37 (<0.001)	151	160	250	17.59 (<0.001)
			(53.7)	(39.6)	(6.8)		(26.9)	(28.5)	(44.6)	
		*N*	1115	1286	372		634	636	1503	
			(40.2)	(46.4)	(13.4)		(22.9)	(22.9)	(54.2)	

**Table 3:** Factors influencing unmet healthcare needs (*N* = 42,698).

Variables	B	OR	95% CI	*p*
Age				
≥85	0.01	1.01	0.90~1.13	0.839
75~84	−0.07	0.93	0.83~1.03	0.2
65~74 (reference)				
Residential area				
Rural	0.29	1.34	1.21~1.47	<0.001
Urban	0.16	1.18	10.08~1.28	<0.001
Metropolitan (reference)				
Living alone				
Yes	0.19	1.21	1.13~1.31	<0.001
No (reference)				
Family income (monthly)				
(US dollars)				
<2000	−0.03	0.92	0.84~1	0.12
2000–4000	−0.29	0.71	0.62~0.81	<0.001
4001–6000	−0.60	0.54	0.42~0.70	<0.001
>6000 (reference)				
Education				
≤Elementary school	0.55	1.73	1.27~2.36	<0.001
High school	0.34	1.4	1.02~1.92	0.034
≥College (reference)				
Basic livelihood subsidy				
Yes	0.57	1.67	1.50~1.83	<0.001
No (reference)				
Activities of daily living				
A little difficult	0.59	1.81	1.68~1.95	<0.001
Difficult	1.23	3.43	2.97~3.96	<0.001
Easy (reference)				
Subjective health				
Good	−0.79	0.45	0.40~0.51	<0.001
Fair	−0.50	0.6	0.55~0.65	<0.001
Poor (reference)				
Diagnosed with hypertension				
Yes	−0.14	0.86	0.81–0.93	<0.001
No (reference)				
Diagnosed with diabetes				
Yes	−0.13	0.87	0.80~0.94	0.001
No (reference)				

OR = Odd ratio; CI = Confidence interval.

## Data Availability

Publicly available data were used in this study. This data can be found here: https://chs.kdca.go.kr/chs/rdr/rdrInfoProcessMain.do (accessed on 20 May 2020).
